# Circulation of *Dirofilaria immitis* and *Dirofilaria repens* Species in Mosquitoes in the Southeastern Part of Romania, Under the Influence of Climate Change

**DOI:** 10.3390/life15101612

**Published:** 2025-10-16

**Authors:** Larisa Ivănescu, Raluca Mîndru, Ilie Bodale, Gabriela-Victoria Apopei, Lavinia Andronic, Smaranda Hristodorescu, Doina Azoicăi, Liviu Miron

**Affiliations:** 1 Departament Clinics, Faculty of Veterinary Medicine, “Ion Ionescu de la Brad” Iasi University of Life Sciences, 700490 Iași, Romania; larisa.ivanescu@iuls.ro (L.I.); raluca.mindru@iuls.ro (R.M.); lavinia.andronic@iuls.ro (L.A.); liviu.miron@iuls.ro (L.M.); 2 Departament Exact Sciences, Faculty of Horticulture, “Ion Ionescu de la Brad” Iasi University of Life Sciences, 700490 Iași, Romania; 3 Department of Preventive Medicine and Interdisciplinarity, Faculty of Medicine, “Grigore T. Popa” University of Medicine and Farmacy, 700115 Iași, Romania; hristodorescu.smaranda@d.umfiasi.ro (S.H.); doina.azoicai@umfiasi.ro (D.A.)

**Keywords:** *Dirofilaria immitis*, *Dirofilaria repens*, mosquito vectors, estimation infection rate, climate projections

## Abstract

Dirofilariosis, a parasitic disease caused by nematodes of the genus *Dirofilaria*, primarily affects dogs but can also infect other carnivores and, more rarely, humans. In Europe, the most commonly involved species are *D. immitis* and *D. repens*, transmitted through the bites of mosquito vectors. This study, conducted in Tulcea County between April and October 2024, aimed to determine the prevalence of *D. immitis* and *D. repens* in mosquitoes. A total of 1507 mosquitoes were collected and grouped into 76 pools, and subsequently molecular analysis was carried out using qPCR. The estimated infection rate (EIR) was calculated using statistical methods available in the ‘binGroup’ package in R, which allow the determination of the point estimate and confidence interval (CI) for a single binomial proportion in group testing. The study revealed a high infection rate with *D. immitis* (48%), while *D. repens* was identified in only two pools. The species with the highest vector potential was *Anopheles maculipennis* (PTP = 75%, EIR = 0.1168 with both *Dirofilaria* species), followed by Aedes vexans. Notably, Aedes albopictus was identified for the first time in Tulcea, and all individuals were positive for *D. immitis*. Simulations of local thermal conditions using the proposed model show that the favorable time window for mosquitoes will increase until 2100. Our results indicate an established and active transmission cycle of *D. immitis* in the region, a situation projected to intensify with climate change requiring urgent monitoring.

## 1. Introduction

Dirofilariosis is a parasitic disease caused by nematodes of the genus *Dirofilaria*, primarily *D. immitis* and *D. repens*, which are prevalent in Europe [1,2]. Both circulate in an enzootic cycle between mosquitoes and domestic dogs, although other carnivores such as red foxes or gray wolves can serve as reservoirs [3,4]. These parasites are transmitted through the bites of infected mosquitoes and affect carnivorous mammals worldwide, primarily dogs. They can also infect cats and, more rarely, humans [3,5,6,7,8,9,10]. In Europe, the disease has become more and more common. It now affects the health of companion animals and, in some cases, also impacts human health [11,12,13,14,15]. There are about 70 known species of Dirofilaria. Human infections are most often caused by *D. immitis*, *D. repens*, and *D. tenuis*, while other species such as *D. striata*, *D. ursi*, *D. spectans*, and *D. magnilarvata* are reported only occasionally. The main natural hosts for these species are dogs and wild canids (*D. immitis* and *D. repens*) and raccoons (*D. tenuis*) [16,17,18,19,20].

Countries previously considered free of *Dirofilaria* are now regarded as endemic [21]. Climate warming is believed to be the main factor allowing nematodes to develop successfully in mosquitoes [22], along with increased dog movement facilitated by European travel regulations for pets [20]. Recently, more cases have been reported in Southeastern Europe, Sri Lanka, and southern India. This highlights the need for molecular testing and large-scale epidemiological studies.

Initially, dirofilariosis was mostly reported in southern European countries like Italy, Spain, Greece, and France [23,24]. However, in the last few decades, it has spread into northern regions such as Germany, Austria, and even southern Sweden [25]. This spread is attributed to climate changes that favor mosquito proliferation, especially *Aedes* and *Culex* species [15,17]. Pet travel and animal migrations have also contributed to spreading the disease in non-endemic areas [26].

In Romania, *Dirofilaria* spp. was first identified about two decades ago [27]. Studies on *Dirofilaria* spp. prevalence in dogs have shown infection rates from 3% up to over 60% [28,29,30,31]. *D. immitis* and *D. repens* have also been found in wild carnivores such as red foxes, golden jackals, weasels, wild cats, and gray wolves. These animals probably act as important reservoirs in natural areas where the disease occurs [7,32]. However, human cases in Romania remain low, possibly due to limited physician awareness concerning this parasite.

To date, research on the circulation of *Dirofilaria* species in Romania has primarily focused on monitoring canine populations, with evidence of parasite circulation documented as early as 2007 [27], whereas vector-related studies have been limited. More recently, Tomazatos et al. (2018) [33] conducted an investigation in the Danube Delta, reporting a *D. immitis* prevalence of 4.53% in mosquito samples (with pools containing up to 250 specimens) and 19.44% in dog blood samples.

*D. immitis* remains largely restricted to southern European countries with Mediterranean climates (Cs—hot & dry summers and cool & wet winters according to Köppen climate classification system) but has gradually expanded north [22]. Most European countries have now become endemic, with significant distribution changes in the last 20 years [34,35]. Predictions suggest dirofilariosis will spread rapidly, urging implementation of monitoring and control programs at the European level [36]. The increase in global trade, travel, and environmental changes in recent decades has greatly contributed to the fast spread of vector-borne diseases [37].

Monitoring dog infections is difficult, as the disease is often asymptomatic or presents mild symptoms, but severe forms can cause congestive heart failure, chronic cough, weakness, and death [38,39]. In humans, *D. repens* infections are more common and may cause subcutaneous nodules, sometimes painful or inflamed. Also it can cause pulmonary inflammation triggered by dead adult worms, visible as coin lesions on X-rays [40,41,42]. However, most human cases are asymptomatic. When symptoms appear, they include cough (sometimes with blood), chest pain, fever, and pleural effusion [35,43]. Rarely, *D. immitis* worms have been found outside the lungs (e.g., brain, eyes, testicles) [11,44]. *D. repens* and *D. tenuis* typically cause subcutaneous nodules or, occasionally, are found in the conjunctiva [45,46,47,48,49,50]. Some reports describe viable *D. repens* microfilariae in human blood [51,52]. Diagnosis involves serological testing, PCR, and blood microscopy. Ultrasound may also detect adult worms in the heart or other organs [53].

Disease spread is favored by mosquito presence, freshwater, high humidity, and warm temperatures. Higher ambient temperatures shorten larval development time in the vector [54]. The number of mosquito species implicated in *Dirofilaria* transmission increases yearly, including both endemic and invasive species spreading due to climate change [9,54]. Currently, at least 77 mosquito species (Diptera: *Culicidae*) from the genera *Culex*, *Aedes*, *Anopheles*, *Mansonia*, *Coquillettidia*, *Psorophora*, and *Culiseta* are considered to act as vectors [55]. Among these, *Aedes vexans* is highly efficient due to its aggressive hematophagy and wide distribution. *Culex pipiens,* adapted to urban and suburban settings, plays a major role in temperate regions [55,56]. *Anopheles* mosquitoes, especially *A. maculipennis*, maintain the transmission cycle in rural areas of Europe and Asia [57,58]. *Ochlerotatus* species, like *O. caspius,* are also competent vectors, particularly in Mediterranean and subtropical regions [59].

## 2. Materials and Methods

(a) Biological material

The objectives of the study aimed to establish the prevalence of *D. repens* and *D. immitis* in mosquitoes from the Tulcea area, as well as to predict the evolution of this prevalence under global warming conditions. Between April and October 2024, mosquito traps were installed to assess the transmission risk of *D. immitis* and *D. repens* in Tulcea County. The collection points selected were: Somova commune, human dwelling in Mineri village (longitude 28°43′27.8″ E, latitude 45°10′10.5″ N), Nufăru commune, human dwelling in Victoria village (longitude 28°57′55.0″ E, latitude 45°10′58.9″ N), veterinary clinic with animal housing in Murighiol commune (longitude 29°09′58.3″ E, latitude 45°02′15.1″ N), and Tulcea city public animal shelter (longitude 28°45′17.1″ E, latitude 45°10′58.9″ N).

CDC Light traps, model 1012 (John W. Hock Company, Gainesville, FL, USA), were used on the first and last weekend of each month during the study period (a total of four traps were employed, all being simultaneously positioned at the predetermined sites). The traps were deployed overnight from 19:00 to 08:00. Mosquito species were identified based on morphological features, using the identification keys by Norbert Becker [59] and the interactive keys from the MosKeyTool v2.1 software (Pasteur Institute, Paris, France). After morphological identification, mosquitoes were stored at −80 °C and grouped into species-specific pools, each pool containing 20 mosquitoes. The pools were divided by species for each location. Since the pool positivity was very similar across all locations, the prevalence of *D. immitis* and *D. repens* was calculated for the entire study area. Pools are made in order to reduce the overall costs; however, care should be taken regarding the size of pooled samples not to exceed the detection limit. Therefore, the smaller the pool, the better chance of identifying positive samples.

(b) Molecular screening

The pools created at the moment of the morphological identification each contain 20 mosquito specimens, as was previously described. We have classified the samples as tissues; therefore, we added an extra step: preincubation of the samples, using 20 μL of proteinase K and 180 μL PureLink™ Genomic Digestion Buffer in each tube, according to the extraction protocol. We set for overnight digestion while incubating at 55 °C with periodic vortexing. Molecular screening began with DNA extraction from pools using the PureLink Genomic DNA Mini Kit (Thermo Fisher Scientific, Waltham, MA, USA). DNA was extracted from 200 µL of digested pool, following the manufacturer’s protocol, and each pool eluate (100 μL) was stored at −20 °C until molecular detection was performed. For pathogen detection, a multiplex qPCR protocol was used, combining TaqMan (*D. immitis* CAT CCT GAG GTT TAT GTT ATT ATT TT, CWG TAT ACA TAT GAT GRC CYC A, and 6FAM-CGG TGT TTG GGA TTG TTA GTG-MGB) and SYBR Green (*D. repens* with TM 70: GTG TGC TGC GCT ACA TCG ATG TT, ATA AAC CGC TCT GTC TCA CGA CG) principles. QuantiNova Multiplex PCR Master Mix 4× (Qiagen, Venlo, The Netherlands) was used in real-time tests performed in a C1000™ thermocycler (Bio-Rad Laboratories Inc., Hercules, CA, USA), using the CFX96™ Real-Time Detection System. Amplification reactions for all qPCRs included 5 μL of QuantiNova Multiplex PCR Master Mix (Qiagen, Venlo, The Netherlands), 5 μL of DNA template, primers (0.4 μM), probes (0.25 μM), and molecular-grade water up to a final volume of 20 μL. The result validation comprised both an endogenous control (Cy5) that targeted a housekeeping gene to evaluate extraction and amplification efficiency, as well as an exogenous internal control (for possible amplification inhibitions)—HEX [60], and also a positive (Ct = 22.3) and negative control.

The thermocycling program consisted of an initial denaturation step at 95 °C for 10 min, followed by 40 cycles of 95 °C for 15 s and 62 °C for 1 min. Melting temperature (Tm) measurements were taken between 65 and 88 °C at 0.5 s intervals [61,62]. Fluorescent signals collected from FAM and HEX channels were analyzed using CFX Manager Software Version 3.1. Primers (Bio-Rad Laboratories Inc., Hercules, CA, USA) recommended by Pękacz [63] were used for *D. repens* detection, and primers recommended by Negron [64] were used for *D. immitis* detection (Table 1).

(c) Estimation infection rate method

In the laboratory, we used the qPCR technique to identify the presence of *D. repens* and *D. immitis* in mosquito samples. In epidemiology, mosquito populations are classified into pools to determine whether they are infected or not [33,57]. Mathematically, we estimated the infection rate in the pool using a statistical method for group testing to determine the probability of positive material in the pool [65]. This method is based on binomial group testing, a complex algorithm typically involving probability models and statistical estimation techniques.

Estimated infection rate and confidence interval (CI) were calculated using the ‘bgtCI’ function from the ‘binGroup’ package in R, which accounts for pooled testing [66]. The proportion of positive pools was calculated according to a 95% confidence level [66].

The proportion of tested pools (PTP) that are positive was calculated using:

$$\mathrm{PTP}=\frac{\mathrm{Number}\;\mathrm{of}\;\mathrm{mosquito}\;\mathrm{pools}\;\mathrm{positive}\;\mathrm{for}\;\mathrm{Dirofilaria}}{\mathrm{Number}\;\mathrm{of}\;\mathrm{total}\;\mathrm{pools}}\cdot 100\;(\%)$$

where PTP (Proportion of Tested Pools that are Positive) is the proportion of mosquito pools positive for *Dirofilaria* species.

The prevalence of *Dirofilaria immitis* and *Dirofilaria repens* infection in the collected mosquitoes was also estimated using the Minimum Infection Rate (MIR), i.e., the minimum proportion of infected mosquitoes expressed as a percentage: MIR = (p/N) × 100%, where p represents the number of positive pools and N is the total number of mosquitoes tested. This method assumes that in each positive pool there is at most one infected mosquito.

(d) Climatic model

In the present study, we analyzed the thermal characteristics of the air near the surface (2 m) during the period 1961–2024, making estimates of long-term temperature changes in order to understand the influence of these changes on development of mosquito populations.

**Figure 1:**
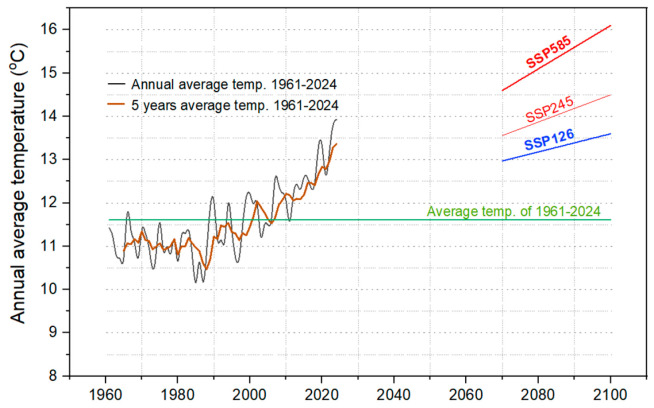
Temperature evolution over the past six decades and projected trends for the last decades of 21st century. The annual (black line) and 5-year (brown line) averages were calculated for diurnal temperatures recorded at a height of 2 m in the Tulcea weather station (1961–2024). Long-term projections were generated using an adapted local version of the CMIP6 climate scenarios model. The three temperature scenarios for 2070–2100 were simulated: (1) SSP126 (blue line)—representing low greenhouse gas emissions (GHGE); (2) SSP245 (thin red line)—representing an intermediate-emission scenario and (3) SSP584 (thick red line)—a high-emission scenario.

We used a simplified and adapted climatic model based on the global climate modeling project CMIP6 (Coupled Model Intercomparison Project Phase 6), taking into consideration the local climatic conditions in Tulcea County (elevation ranging from sea level up to 467 m). The CMIP6 estimates the air temperature near the surface (at 2 m) based on the heat balance determined as radiative forcing (RF) of the air using the three possible scenarios: SSP126 (RF_SSP126_ = 2.6 W/m^2^), SSP245 (RF_SSP245_ = 4.5 W/m^2^) and SSP585 (RF_SSP585_ = 5.8 W/m^2^). In this approach, the temperature projections are made by considering different hypothetical scenarios given by the air carbon dioxide concentrations. The NetCDF output files from CMIP6 archives estimate surface air temperatures (‘tas’ variable) on long term until 2100 by using the IPSL–CM6A–LR climatic model [67]. We identified the fitting parameters of *tas* variables that range from IPSL–CM6A–LR to obtain the fingerprint of global warming curve. The fitting parameters were used for Tulcea temperature range to simulate the values for long-term 2070–2100 (Figure 1). In proposed model, the input data used was the range of average daily temperatures measured during the period 1961–2024 at Tulcea weather station from Romania (STAID: 967 from the European Climate Assessment & Dataset—ECA&D).

(e) Heartworm Development Unit

The heartworm development unit (HDU) is a bioclimatic index that establishes the existing incubation periods of *Dirofilaria* spp. based on accumulated temperature. Studies show that the minimum biological temperature threshold for the development of *Dirofilaria* spp. in mosquitoes is an average diurnal temperature (*T_d_*) of 14 °C. From thermal point of view, the population of mosquitoes passes from microfilaria (L1) to the infective (L3) stages, it is necessary to accumulate 130 HDUs in less than 30 days [68]:

$$\mathrm{HDU}=\sum_{i=1}^{30}\min\{\max[(T_{d}\;-\;14),0],\;(40\;-\;T_{d})\},$$

where 14 is the temperature of 14 °C, which is heartworm development threshold (*T_d_* ≥ 14 °C), and 40 (°C) is the heartworm development ceiling (*T_d_* ≤ 40 °C) for *Dirofilaria* spp., but such high temperatures are not reached in Romania.

## 3. Results

A total of 1507 mosquitoes, identified to species or complex level based on morphological criteria, were tested for the presence of *Dirofilaria* spp. Pools were created with the species *Aedes caspius*, *Aedes vexans*, *Aedes albopictus*, *Culex pipiens s.s./Cx. torrentium*, *Anopheles hyrcanus*, *Anopheles maculipennis s.l.*, and *Uranotaenia unguiculata,* with 20 specimens included in each pool. In total, 76 pools were tested (Table 2).

The pools were classified according to the seven mosquito species sampled from the studied area (pooling by species was performed across all collection sites, as the samples were processed fresh and in order to ensure a consistent number of specimens per pool): *Uranotaenia unguiculata*, *Aedes albopictus*, *Aedes vexans*, *Anopheles hyrcanus*, *Anopheles maculipennis s.l.*, *Culex pipiens s.s./Cx. torrentium* and *Aedes caspius*. The *Culex pipiens s.s./Cx. torrentium* was the most numerous (580 mosquitoes), while *Aedes albopictus* was the least numerous (7 mosquitoes) (Table 2). The small number of *Aedes albopictus* identified in the field means the data are not statistically significant, allowing us to only indicate the presence of *Dirofilaria immitis* in this potential competent vector. We specify that this is the first report of the species *Aedes albopictus* in Tulcea County, with the nearest place where it has been previously reported being the Great Brăila Island in 2023 [69]. The 48% PTP value for all examined species raises an alarm that climate change is having a significant impact, and, in the absence of monitoring and vector control measures, the risk of *Dirofilaria immitis* infection has reached a very high level.

Within the 76 mosquito pools tested in the laboratory using the qPCR technique to identify *Dirofilaria* species DNA sequences, a high rate of positive infections (39 pools) was identified. The proportion of positive *D. immitis* pools was very high for all tested samples (PTP > 29%) but low for *D. repens* infection (PTP = 16.67%) in all the mosquito populations tested.

The average infection probability for each pool of *Dirofilaria* is EIR = 0.03406, with a confidence interval (CI) distribution of [0.02489, 0.04682]. The mosquito species with the highest probability of being a vector for both *Dirofilaria* species is *Anopheles maculipennis*, with an estimated infection probability of EIR = 0.1168 per pool and a CI = [0.05061, 0.1898] at a 95% confidence level. In contrast, *Anopheles hyrcanus* is the least vulnerable species to becoming a vector for *Dirofilaria* spp. (EIR = 0.01033; CI = [0.00340, 0.02772]).

We can state that *Anopheles maculipennis* shows the highest importance as a vector for *Dirofilaria immitis* and *Dirofilaria repens*, with a PTP value of 75%, followed by *Aedes vexans* with a PTP of 50%. For *Aedes albopictus*, we can only indicate the presence of *Dirofilaria immitis*, since only one pool consisting of seven specimens was analyzed, but we emphasize the vectorial potential and the rapid spread of this invasive species in Romania.

All mosquito species identified in the field had individuals infected with *D. immitis* (Table 3), but *D. repens* was only identified in *Anopheles maculipennis* pools (Table 4).

Based on the MIR calculation formula, the total prevalence for *Dirofilaria immitis* is estimated to be 2.38%. The prevalence is 1.66% for *Uranotaenia unguiculata*, 14.28% for *Aedes albopictus*, 2.5% for *Aedes vexans*, 1.87% for *Anopheles hyrcanus*, 3.75% for *Anopheles maculipennis*, 2.06% for the *Culex pipiens* complex, and 1.42% for *Aedes caspius*. However, given the small and fixed number of mosquitoes per pool and the low CT values (ranging from 17.25 to 29.34), which indicate a high concentration of *D. immitis* DNA, we consider that this method does not accurately reflect the reality.

Climatically, Tulcea County is in a warm continental climate region (Dfa-Köppen climate classification), with warm oceanic climate influences (Cfa) in the coastal area of the Black Sea, without a dry season (ANM, 2008). Instead, our simulation using proposed climatic model highlights that average temperature will increase somewhere between 13.6 °C (low emissions—SSP126) and 16.1 °C (high emissions—SSP585) in 2100 (Figure 1).

The HDU over multiple decades from 1961 to 2020 was calculated to identify the periods favourable for development of *Dirofilaria* spp. from the microfilaria (L1) to the infectious stage (L3). On average, the temperature supports the start of microfilaria development on May 9 (1961–2020), with the first generation reaching the L3 stage by approximately June 6. However, the date after which temperatures drop below 14 °C has changed from October 30 in 1961–1990 decade to November 3 in 1991–2020 decade.

**Figure 2:**
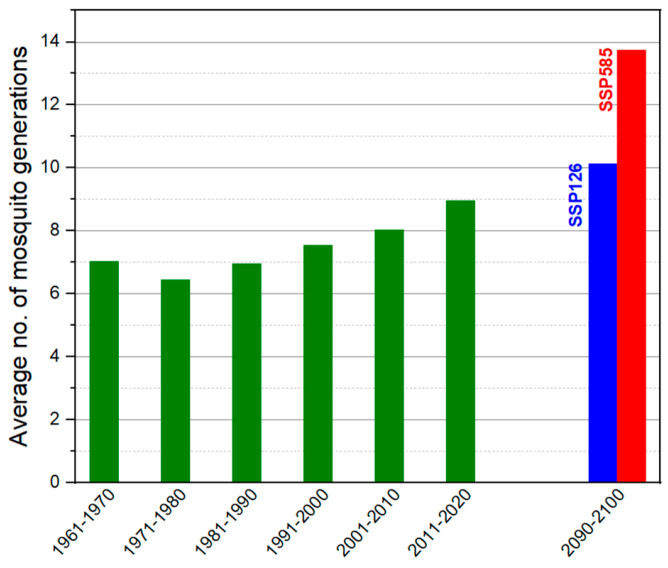
The possible average number of mosquito generations that could develop under the temperature conditions in each decade from 1961 to 2020, and the estimates of the number of generations that will possibly develop in 2091–2100 decade. The estimations were calculated based on long-term temperature projections under the two extreme scenarios (SSP126 and SSP585) of CMIP6 climate model.

Climate-based forecasting systems typically use the concept of growing degree days (GDD), which represent the accumulation of heat units when the average daily temperature exceeds the threshold temperature by 1 °C. The seasonal transmission model of *Dirofilaria* spp. assumes that 130 HDUs must accumulate for the larvae to reach the infective stage, with a maximum lifespan of 30 days for the mosquito vector. Simulations of local thermal conditions using the proposed climatic model show that the favourable time window for mosquitoes developed will extend until the year 2100. The average annual number of *Dirofilaria* spp. generations will increase from 8.17 in 2020 to 13.75 by the end of the century (Figure 2).

## 4. Discussion

A systematic review conducted in 2025 by Hattendorf and Lühken [20], which analyzed 3.847 publications, highlighted that Romania and Bulgaria have a large number of stray dogs, which serve as a significant reservoir for parasites. These countries also report a faster development of *Dirofilaria* spp. larvae within mosquitoes. The spread of *Dirofilaria* infestations had already been predicted in the early 2000s [2,68]. Moreover, rising temperatures in Europe have facilitated the widespread establishment of exotic, diurnal mosquito species such as *Aedes albopictus*, a major vector for *D. immitis* and *D. repens*. Consequently, the only tested pool in our study was found to be positive for *D. immitis*.

To date, studies on the circulation of *Dirofilaria* species in Romania have focused mainly on monitoring dogs, with evidence of parasite circulation reported as early as 2007 [27], while studies focusing on vectors have received less attention. More recently, Tomazatos et al. (2018) [33] conducted a study in the Danube Delta and reported a prevalence of 4.53% for *D*. *immitis* in mosquitoes (the pools were large, with up to 250 mosquito specimens per pool) and 19.44% in dog blood samples. In contrast, our study highlighted a prevalence of 48% for *D*. *immitis* in mosquitoes, which aligns with data reported in dogs in the eastern part of Romania, where a prevalence of 60% was recorded in 2016 [70], and a recent study conducted in the same area as ours reported a prevalence of 50% for *D. immitis* [71].

Molecular detection of filarioid parasites in hematophagous vectors remains the most efficient method for assessing the prevalence of both vectors and pathogens in specific regions. This approach has been used in most studies reporting the prevalence of *D. immitis* and *D. repens* [72,73].

It is very difficult to determine the number of mosquitoes infected with *Dirofilaria*, as this requires significant human, material and financial resources. The binomial group testing statistical method used by us is accurate and efficient because it reduces testing time and costs compared to individual testing [74]. This method statistically analyses the probability of infection in a pool by calculating two parameters (point estimation and confidence interval) of the distribution function [75]. The ‘bgtCI()’ function used in the present study is based on the Wilson score, which provides accurate confidence intervals for small pool sizes (20 mosquitoes).

Previous studies in the Danube Delta and neighbouring areas confirm that *Anopheles maculipennis* and *Aedes* spp. have the highest *Dirofilaria* spp. infection rates [56,64], most likely due to specific local environmental conditions. Several studies have highlighted that the species of mosquito responsible for transmitting infection is influenced by the size of the mosquito population and the density of microfilariae in the blood of hosts [76], which is closely related to urbanisation, deforestation, pollution and organic matter [77,78], as well as climatic conditions [57].

Climatic conditions are important in this type of study because temperature and humidity are essential for mosquito colony development. Tulcea County is a special area because approximately 40% of its territory is covered by Danube Delta, which represents a favourable place for the development of mosquito populations. Our simulations, based on an adapted CMIP6 model and local conditions, highlight that thermal conditions will change locally, extending the mosquito development period to the end of autumn till the year 2100. This will increase the incidence of *Dirofilaria* spp. infection.

The results of this study highlight a high prevalence of *D. immitis* infection in mosquito populations collected from Tulcea County between April and October 2024, with a pool positivity rate of 48%. The species with the highest vectorial potential were *Anopheles maculipennis* (PTP = 75%) and *Aedes vexans* (PTP = 50%), both known for their adaptability to the region’s wet habitats. In contrast, *D. repens* was identified in only two pools belonging to the species *Anopheles maculipennis*, suggesting a lower circulation of this pathogen in the region compared to *D. immitis.* A study conducted for Europe shows that most reports identify species from the *Culex pipiens* complex and *Anopheles maculipennis* as the main vectors for *D. immitis* and *D. repens*, in agreement with our results [20].

The average estimated EIR value for all pools was 0.03217, with confidence intervals indicating a consistent probability of infection among the investigated species. Among them, *Anopheles maculipennis* showed the highest likelihood of being a vector (EIR = 0.1168), positioning it as a key species in the transmission of dirofilariosis in the area. *Aedes albopictus* was reported for the first time in Tulcea County, indicating the rapid spread tendency of this species in Romania. Only seven specimens were identified, and the single pool formed tested positive, suggesting a significant vector potential that requires further validation through subsequent collections.

From a climatic perspective, the analysis of air temperature evolution from 1961 to 2024 indicates a significant increase in the annual average, from 11.6 °C to forecasted values of up to 16.1 °C under the pessimistic SSP585 scenario. This trend is also confirmed by the analysis of the Heartworm Development Unit (HDU) index, which shows an extension of the favorable window for the development of *Dirofilaria* spp. larvae in vectors, both in terms of duration and number of generations. Our simulation suggests an increase in annual generations from an average of 8.17 at present to approximately 13.75 by the year 2100, implying a significant intensification of vector-borne transmission risk in the context of global warming.

The mosquito population in Tulcea/Dobrogea in 2024 had a very high transmission rate of *Dirofilaria*. *D. immitis* was predominant, with *Anopheles maculipennis* being the most important vector. The estimated infection rate of *Anopheles hyrcanus* is 10 times lower than that of *Anopheles maculipennis*.

The exceedance of the annual mean temperature by 2 °C—a threshold that can trigger the development of *Dirofilaria immitis* within mosquitoes—and the rise to an average of 16 °C in 2024 sound an alarm regarding the accelerated increase in the risk of transmission of this disease across the country. This highlights the urgent need to implement vector monitoring and control programs, as well as prophylactic measures among dogs, including those in shelters. If the current situation is already alarming due to the accelerated risk of parasite transmission—with a marked difference in the prevalence of *Dirofilaria immitis* in mosquitoes compared to the last study conducted in the region [33]—the outlook for the future is even more concerning, as a significant increase in the number of parasite generations is projected, from 8.17 to 13.75 by the year 2100. This study issues a clear warning, emphasizing the danger posed to both public health and veterinary medicine by the rapid spread of *Dirofilaria* spp.

*D. repens* infection was detected at a significantly lower rate. The discovery of the species *Aedes albopictus* in Tulcea County represents a noteworthy observation and suggests a possible expansion of the range of this invasive species. Climate models indicate a significant extension of the period favorable for the transmission of dirofilariosis by vectors until the year 2100, in correlation with the increase in average annual temperatures, which supports the need to implement proactive entomological and epidemiological monitoring measures in the region, as well as to develop integrated vector control strategies adapted to changing climatic conditions, while continuously taking into account the predictions made using accepted mathematical models.

## 5. Conclusions

The proportion of positive *D. immitis* pools was very high for all tested samples (PTP > 29%) but low for *D. repens* infection (PTP = 16.67%) in all the mosquito populations tested, with 75% PTP for *Anopheles maculipennis* and 50% PTP for *Aedes vexans,* emphasizing the important role of these two species as competent vectors. The species *Aedes albopictus* was also reported; however, due to the small number of specimens collected, we can only indicate the presence of *Dirofilaria immitis* and highlight the need for continued monitoring of this species in the future. The mathematical models used indicate a future increase in the number of *Dirofilaria* spp. generations developing annually within mosquito vectors, from 8.17 to 13.75, emphasizing the necessity of implementing control programs that are always correlated with ongoing climate changes.

The results of the study raise an alarm both in human and veterinary medicine. Considering that stray dogs represent a major problem in Romania, they remain the main natural reservoir for dirofilariosis. Therefore, the administration of prophylactic treatment becomes mandatory for both shelter dogs and dogs with owners. Moreover, the findings of our study should serve as an important criterion in the differential diagnosis of respiratory conditions involving pulmonary masses, prior to proposing any invasive procedure.

## Figures and Tables

**Table 1:** Primers used for RT-PCR detection of *D. repens* and *D. immitis*.

Species	Principle	Primer/Probe	Sequence 5–3′	Gene Target	References
*Dirofilaria repens*	SYBR Green	For_s16	GTG TGC TGC GCT ACA TCG ATG TT	16S rRNA	[62]
		Rev_s16	ATA AAC CGC TCT GTC TCA CGA CG		
*Dirofilaria immitis*	TaqMan	Fil.COI.749-F	CAT CCT GAG GTT TAT GTT ATT ATT TT	Cox-1	[63]
		Fil.COI.914-R	CWG TAT ACA TAT GAT GRC CYC A		
		Probe	6FAM-CGG TGT TTG GGA TTG TTA GTG-MGB		

**Table 2:** Results of pool-based identification of *D. immitis* and *D. repens*.

Mosquito Species	Total Pools Tested (20 Mosquitoes/Pool) *D. immitis* and *D. repens*	*D. immitis* (Positive Pools)	*D. repens* (Positive Pools)
*Ur. unguiculata*	3	1	0
*Ae. albopictus*	1	1	0
*Ae. vexans*	16	8	0
*An. hyrcanus*	8	3	0
*An. maculipennis*	12	9	2
*Cx. pipiens complex*	29	12	0
*Ae. caspius*	7	2	0
**Total**	**76**	**36**	**2**

**Table 3:** Statistical evaluation of the probability of *D. immitis* infection in mosquito species sampled in Tulcea area in 2024.

Mosquito Species	No. of Mosquitoes	No. of Total Pools	No. of Pools Positive Tested for *D. immitis*	PTP (%)	EIR	CI
					for Confidence Level by 95%	
*Ur. unguiculata* *	60	3	1	33%	0.02007	0.00317–0.07558
*Ae. albopictus* **	7	1	1	100%	1	0.03251–1
*Ae. vexans* *	320	16	8	50%	0.03406	0.01629–0.06167
*An. hyrcanus* *	160	8	3	38%	0.01033	0.00341–0.02772
*An. maculipennis* *	240	12	9	75%	0.06697	0.03104–0.11400
*Cx pipiens complex* *	580	29	12	41%	0.02635	0.01462–0.04391
*Ae. caspius* *	140	7	2	29%	0.01668	0.00428–0.04994
Total *	**1507**	**75**	**36**	**48%**	**0.03217**	**0.02289–0.04375**

* The total number of mosquitoes in pools is 20, ** except for the pools *Aedes albopictus* where there are 7 mosquitoes.

**Table 4:** Statistical evaluation of the probability of *D. repens* infection in mosquito species sampled in Tulcea area in 2024.

Mosquito Species	No. of Mosquitoes	No. of Total Pools	No. of Pools Positive Tested for *D. repens*	PTP (%)	EIR	CI
					for Confidence Level by 95%	
*Ur. unguiculata* *	60	3	0	0.0	0.0	0.0–0.04038
*Ae. albopictus* **	7	1	0	0.0	0.0	0.0–0.20170
*Ae. vexans* *	320	16	0	0.0	0.0	0.0–0.01070
*An. hyrcanus* *	160	8	0	0.0	0.0	0.0–0.01942
*An. maculipennis* *	240	12	2	16.67	0.00079	0.00022–0.00283
*Cx pipiens complex* *	580	29	0	0.0	0.0	0.0–0.00620
*Ae. caspius* *	140	7	0	0.0	0.0	0.0–0.02164
**Total** *	**1507**	**75**	**2**	**2.67**	**0.00135**	**0.00037–0.00482**

* The total number of mosquitoes in pools is 20, ** except for the pools *Aedes albopictus* where there are 7 mosquitoes.

## Data Availability

Data are contained within the article.
